# An evaluation of Comparative Genome Sequencing (CGS) by comparing two previously-sequenced bacterial genomes

**DOI:** 10.1186/1471-2164-8-274

**Published:** 2007-08-14

**Authors:** Christopher D Herring, Bernhard Ø Palsson

**Affiliations:** 1 Department of Bioengineering, University of California San Diego, La Jolla, USA; 2 Mascoma Corporation, 16 Cavendish Court, Lebanon, NH 03766, USA

## Abstract

**Background:**

With the development of new technology, it has recently become practical to resequence the genome of a bacterium after experimental manipulation. It is critical though to know the accuracy of the technique used, and to establish confidence that all of the mutations were detected.

**Results:**

In order to evaluate the accuracy of genome resequencing using the microarray-based Comparative Genome Sequencing service provided by Nimblegen Systems Inc., we resequenced the *E. coli *strain W3110 Kohara using MG1655 as a reference, both of which have been completely sequenced using traditional sequencing methods. CGS detected 7 of 8 small sequence differences, one large deletion, and 9 of 12 IS element insertions present in W3110, but did not detect a large chromosomal inversion. In addition, we confirmed that CGS also detected 2 SNPs, one deletion and 7 IS element insertions that are not present in the genome sequence, which we attribute to changes that occurred after the creation of the W3110 lambda clone library. The false positive rate for SNPs was one per 244 Kb of genome sequence.

**Conclusion:**

CGS is an effective way to detect multiple mutations present in one bacterium relative to another, and while highly cost-effective, is prone to certain errors. Mutations occurring in repeated sequences or in sequences with a high degree of secondary structure may go undetected. It is also critical to follow up on regions of interest in which SNPs were not called because they often indicate deletions or IS element insertions.

## Background

Genome resequencing is the determination of a genome sequence using an already established genome sequence as a reference. In hybridization-based resequencing, the reference is necessary for the generation of microarray probes and for signal normalization. In other types of resequencing the reference is used as a scaffold for the assembly of short sequence reads. The genome to be resequenced must be substantially similar to the reference; otherwise the reference looses its effectiveness.

Resequencing is useful for relating phenotype to genotype and for analyzing natural variation. It has been used to study the acquisition of antibiotic resistance [1,2], the analysis of variation in pathogenic bacteria and viruses [3-7], and to study the experimental evolution of bacteria and yeast [8-11]. Methods of resequencing utilize microarrays [2,7,10], polonies [9] or sequencing-by-synthesis technology [12]. The Comparative Genome Sequencing service provided by Nimblegen Systems Inc. is a hybridization-based method and consists of two steps [2]. In the first step, an experimental and reference genomic DNA sample are labeled and hybridized to microarrays containing ~30-mer 'tiled' oligonucleotides spaced every ~7 bp of the genome on both strands. Probes cover every nucleotide of the genome and are designed to have isothermal hybridization characteristics. Probes showing differences in signal intensity are flagged as Regions Of Interest (ROIs) for further investigation. A second microarray is then designed to interrogate the ROIs at single base-pair resolution, with all four possible nucleotides synthesized for each interrogated position on both strands (8 probes per position). If the sequence difference is a single-base polymorphism, the pattern of probe intensities at that position will be discernibly different than expected. Other types of sequence differences such as insertions/deletions (indels) or differences of more than one bp will not be determined conclusively.

In order to attribute phenotype to a genotype it is important to know that all relevant sequence differences were detected. False negatives (i.e. failures to detect mutations that are actually present) can result in erroneous interpretation, especially considering that a single mutation can have a dramatic impact on phenotype. False positives on the other hand (i.e. the reporting of sequence differences that are not actually present) are not a big problem in most cases since even 100 false mutations can be checked and refuted with PCR amplification and Sanger sequencing for less money than a typical resequencing experiment.

In order to determine the false negative and false positive rates for CGS, we utilized two related strains of *E. coli *for which high-quality genome sequences exist. Strain W3110 was resequenced with strain MG1655 as the reference, but this setup could just as easily have been reversed. Our results demonstrate the accuracy of CGS resequencing but also raise caveats about CGS and the instability of bacterial strains.

## Results

The closely related and fully sequenced *E. coli *strains W3110 and MG1655 represent an excellent test for the accuracy of resequencing technology. Hayashi et al. [13] compared the genome sequences of these two strains and resolved all discrepancies, generating a pair of highly accurate genome sequences. The genome sequence of strain W3110 differs from MG1655 by seven single nucleotide polymorphisms (SNPs), one 2 bp insertion, 12 IS element insertions, one deletion of 6.6 kb, and an inversion of 783.1 kb [13]. A summary of all sequence differences between strain MG1655 and W3110 is presented in Table 1.

**Table 1:** Differences between MG1655 and W3110 Kohara (CGSC # 7167)

Location^1^	Gene	b number	Mutation	CGS Detection	Reported as:^2^	Status^3^
Small sequence differences						

555954	*ybcJ*	b0528	c>t	yes	SNP	new
556858	*folD*	b0529	a>t	yes	SNP	new
1092487	*ycdT*	b1025	c>t	yes	SNP	known
1335418	*acnA*	b1276	a>g	yes	SNP	known
1650355	*intQ*	b1579	t>c	yes	SNP	known
2030447	*yedJ*	b1962	g>a	yes	SNP	known
2865477	*rpoS*	b2741	g>a	yes	ROI	known
3484227	*crp*	b3357	c>a	yes	ROI	known
4210321	*rrlE*	b4009	g>a	undetected		known
4364615	*dcuA*	b4138	2 nt insertion	yes	ROI	known
						
IS element insertions						

654214	*dcuC*	b0621	IS5 insertion	yes	ROI	known
1104604	*csgC-ymdA*	b1043 – 1044	IS2 insertion	undetected		known
1298687	*ychE-oppA*	b1242 – 1243	IS2 insertion	undetected		known
1463456	*ydbA*	b1401	IS2 insertion	yes	ROI	new
1976519	*flhD-yecG*	b1892 – 1895	IS5 insertion	undetected		known
2033459	*yedS – hchA*	b1966 – 1967	IS5 insertion	yes	ROI – 1 probe	new
2172870	*gatA*	b2094	IS5 insertion	yes	ROI	known
2315483	*rcsC*	b2218	IS2 insertion	yes	ROI	known
2404811	*lrhA-yfbQ*	b2289 – 2290	IS1 insertion	yes	ROI – 1 probe	known
2696426	*yfhB*	b2560	IS30 insertion	yes	ROI	new
3260500	*tdcD*	b3115	IS5 insertion	yes	ROI – 1 probe	known
3378961	*degQ*	b3234	IS2 insertion	yes	ROI	new
3550943	*malP-malT*	b3417 – 3418	IS5 insertion	yes	ROI	new
3681321	*dctA*	b3528	IS30 insertion	yes	ROI	new
3886653	*tnaA-tnaC*	b3707 – 3708	IS5 insertion	yes	ROI – 1 probe	known
3889134	*tnaB*	b3709	IS5 insertion	yes	ROI	known
3891777	*yieE-yidZ*	b3711 – 3712	IS2 insertion	yes	ROI	known
4229085	*yjbC*	b4022	IS2 insertion	yes	ROI	new
4304907	*alsK*	b4084	IS5 insertion	yes	ROI	known
						
Deletions/Inversions						

1395267 – 1397616	*ynaJ-fnr*	b1332 – 1334	deletion	yes	ROI	new
~2556721 – 2563354	*intZ-yffS*	b2442 – 2450	deletion	yes	ROI	known
3424826 – 4207943		b3276 – 4008	Inversion	undetected		known

1. Location is given based on the MG1655 genome (GenBank accession # U00096.2)

2. Detected differences were reported by Nimblegen as Single Nucleotide Polymorphisms (SNPs) or in non-called Regions Of Interest (ROIs). Most differences detected in ROIs were reported by multiple nearby probes, but were reported in only one probe in cases as noted.

3. This column indicates whether the sequence difference is present in the published genome sequence of W3110.

To test the accuracy of CGS, *E. coli *strains W3110 Kohara and MG1655 were obtained from the E. Coli Genetic Stock Collection (CGSC). DNA was extracted and submitted to Nimblegen Systems Inc. for CGS, using MG1655 as the reference. Nimblegen reported 25 SNPs in strain W3110, 4 of which corresponded to known sequence differences. Regions surrounding the other SNPs were PCR amplified and subjected to Sanger sequencing. In this way, 19 of the remaining putative SNPs were refuted, though 2 were confirmed. These two mutations are not present in the W3110 genome sequence, and must have been introduced into the strain after Kohara et al. [14] made the lambda phage library that was sequenced. In sum, there were 19 false positive SNPs out of 25 reported, or one false positive per 244 kb (Table 2).

**Table 2:** Summary of false negatives/false positives.

Mutation type	No. of real differences^a^	No. detected	False negative rate	No. false positive	False positive rate per 10,000 bp
Small sequence differences	8	7	12.50%	19	0.041
IS-element insertions	12	9	25%	15^b^	0.032
Deletions/inversions	2	1	50%	15^b^	0.032

a. This number only includes those differences reported in reference 13, and not the new ones discovered in this study.

b. The number of non-called ROI's that did not contain mutations was taken as the number of false positives for both IS-element insertions and indels.

**Figure 1:**
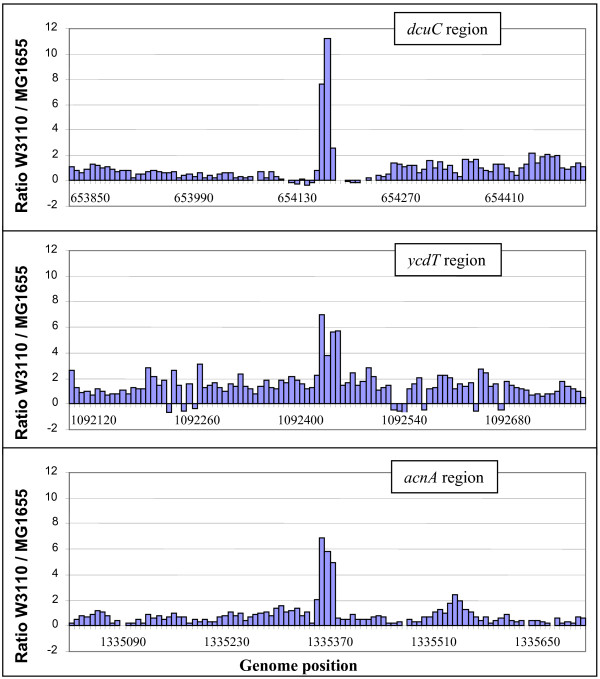
Sample mutation mapping data. The ratio of signal of W3110 vs. MG1655 is shown for probes spaced every ~7 bp surrounding an IS5 insertion in *dcuC *(top), a SNP in *ycdT *(middle), and a SNP in *acnA *(bottom).

Figure 1 shows a sample of hybridization signals surrounding three different sequence differences. It can be seen that the ratio of signal intensities near the mutations were distinctly elevated. When these regions were resequenced at single bp resolution, the sequence differences in *ycdT *and *acnA *were correctly determined. The intensity of hybridization signals in other areas where there were no sequence differences varied considerably.

In addition to SNPs, Nimblegen also provided the location of probes that showed hybridization differences, yet could not be resolved as SNPs ("non-called Regions Of Interest" or ROIs). These probes may indicate the presence of mutations other than SNPs, such as insertions or deletions. Nimblegen reported 1094 ROI probes located in 36 clusters, a cluster being a group of probes that are within 500 bp of each other. One of these clusters corresponded to the known 6.6 kb deletion of *intZ *thru *yffS *(b2442–2450) (Table 1). Three other clusters corresponded to known sequence differences that were not reported as SNPs – the substitutions in *rpoS *and *crp *and the 2 bp insertion in *dcuA*. Nine clusters corresponded to known IS-element insertions in W3110. Most of the IS-element insertions were evident as clusters consisting of multiple ROI probes, though three were only evident as single-probe clusters. There were three IS element insertions that were not detected as either SNPs or ROIs. The large inversion in W3110 of all genes between ribosomal RNA genes *rrlD *and *rrlE *was not detected as either SNPs or ROIs.

One novel cluster of ROI probes was very large, indicating the possible deletion of three genes, *ynaJ*, *uspE*, and *fnr *(b1332–1334). Such a deletion is not present in the genome sequence of W3110. PCR amplification using primers located in adjacent genes confirmed the deletion; a 3.8 kb PCR product was obtained from MG1655 while a 1.4 kb product was obtained from W3110. Deletions of *fnr *have been noted previously in strains of MG1655 obtained from the CGSC [16]. To determine if the deletion occurred before or after the strain was deposited at CGSC, a culture was obtained from the original lyophil made when stock # 7167 was deposited in 1990 by Akira Ishihama. PCR amplification with the same primers showed two products, one at 1.4 kb and another at 5 kb. Sequencing the ends of the 5 kb band revealed the known IS5 element b1331 to the right of *ynaI *on one side and a new IS5 element inserted to the left of *ogt *(b1335) on the other. This result seems to indicate that the strain of W3110 deposited with CGSC has two copies of IS5 on either side of *ynaJ*, *uspE *and *fnr *that undergo recombination with each other at high frequency leading to the deletion of the intervening genes.

The remaining 22 clusters of ROI probes were PCR amplified and Sanger sequenced to see if they indicated additional mutations not present in the W3110 genome sequence. Indeed, seven IS-element insertions were discovered, while the other 15 clusters showed no mutations. We note that all but one of the false positive ROIs were single-probe clusters and that all of them contained inverted repeats of between 6 and 13 nt. These repeats may lead to hairpin structures and poor hybridization properties of those probes [15]. In total, 10 mutations (2 SNPs, 7 IS-insertions and 1 deletion) accumulated in strain W3110 in the time period between when Kohara et al. generated the lambda library and when we obtained it from CGSC. The history and handling of the strain before Ishihama deposited it with CGSC in 1990 is not known by the authors of this study, though we speculate that it may have been stored as a stab at room temperature.

## Discussion

In this study, we sought to evaluate the accuracy of Nimblegen's microarray-based CGS resequencing technology so that the results obtained from experimental samples can be interpreted judiciously. Our results indicate that the false positive rate for SNPs was one per 244,193 bp of sequence. The false positive rate for clusters of ROI probes was one per 309,312 bp of sequence. These rates have been shown to depend on the thresholds chosen for mutation-calling [17]. In theory, low thresholds should be used for mutation mapping, increasing the number of false positives but reducing the number of false negatives. The methods used by Nimblegen to identify ROI's and to determine sequence differences have been described elsewhere [2][18]. ROIs are identified by discarding erroneous probes then picking probes for which the ratio on both strands exceeds 3.5 standard deviations of the 80^th ^percentile within a 1800 bp local window [2]. For sequence determination, Molla et al. [18]. describe a machine-learning algorithm that uses relative positions within "feature space" rather than a training set and describe the effects of changing thresholds on sensitivity. In future work, false positive rates might be reduced by additional development of these algorithms and discarding single-probe clusters containing inverted repeats. Error analysis for development of improved algorithms or array designs should take into account whether errors occurred in the first "mapping" step or the second single bp resolution step of CGS.

A high false discovery rate is generally not a problem with resequencing projects. The cost of PCR amplifying and Sanger sequencing up to 100 candidate regions is small compared to the cost of resequencing itself. The false negative rate on the other hand is very important. In order to associate genotypic change to phenotypic change it is critical to know with some certainty that all of the important mutations were detected. Nimblegen estimates that the false negative rate for SNPs is less than 5% (Tom Albert, personal communication). We found that only half of the small sequence differences actually present were reported as SNPs. If we consider detection in ROI probes as well, then 7 out of 8 small sequence differences were detected, yielding a false negative rate of 12.5%. Given the small sample size, this rate may be consistent with Nimblegen's claims. CGS is meant for the detection of SNPs, but ROI data also reveals deletions and IS-element insertions. The false negative rate for detection of IS element insertions was double the rate for small sequence differences, possibly because CGS has not been optimized for detection of insertions. Conceptually, insertions and inversions could result in a more pronounced hybridization difference at the insertion site. If the insertion site/inversion breakpoint occurs in the middle of the region covered by a probe, then genomic DNA will only match one half of the probe or the other. Detection of insertions might be improved by manipulation of the hybridization/wash conditions or changes to the algorithm to specifically identify probe series indicative of insertions.

The single SNP not detected is a substitution in the gene *rrlE*, which is duplicated 6 other times in the *E. coli *genome. In previous work, we noted the failure of CGS to detect a 9 bp duplication, a 1 bp deletion and a 28 bp deletion near a transcriptional terminator [8]. It appears that CGS has trouble in regions of high local secondary structure and with sequences repeated multiple times in the genome [15][17]. In addition, detection of SNPs may be less accurate in areas with low overall intensity. Fortunately, such regions are relatively uncommon, and CGS appears to detect most mutations with a reasonable degree of confidence.

Site-directed mutagenesis can overcome uncertainty caused by the possibility of false negatives. In our previous work [8], we introduced all of the mutations identified into the wild type strain and were able to reconstruct the observed change in growth phenotype in 4 of 5 cases, indicating that all of the important mutations were found in most cases. An additional precaution against false negatives is resequencing multiple replicates from the same experimental setup. Our previous work identified mutations in the gene *glpK *in 4 of the 5 replicates. Sequencing *glpK *from the one remaining replicate with other technology showed a 9 bp duplication undetected by CGS. This demonstrates the value of replicates and why genes containing mutations in some replicates should be screened for false negatives in the other replicates.

Resequencing technology allows a sequence to be determined at less than one tenth the cost of traditional Sanger sequencing, but it is less accurate with some types of sequences and requires a closely related reference sequence. Other methods of resequencing [9,12] are likely to differ from CGS in cost and accuracy. The control experiment presented here comparing *E. coli *strains W3110 and MG1655 can now be performed with these other technologies to allow cross-comparison.

## Conclusion

We determined the false positive rate for CGS to be one per 244 Kb for SNPs and one per 309 Kb for non-called ROIs. We observed a false negative rate of 12.5% for small sequence differences and 25% for IS-element insertions. The one large deletion present in the genome sequence was easily detected, though the chromosomal inversion was not. We conclude that the accuracy of CGS is sufficient for effective resequencing studies, but with some precautionary notes. All clusters of non-called ROI probes should be PCR amplified and Sanger sequenced to detect IS-element insertions and small indels. Also, multiple replicates and/or site-directed mutagenesis should be used in cases where rare false negatives may affect the scientific interpretation.

We made the unexpected discovery of 10 mutations accumulated in strain W3110 after Kohara et al. generated the lambda clone library that was sequenced. This highlights the ability of CGS to reveal unexpected results and the rapid degeneracy of strains under some kinds of storage conditions. Strains with the same name (e.g. W3110, MG1655) often differ depending on their source and storage conditions, so sequenced strains should only be obtained from the laboratories that sequenced them or their designated depositories.

## Methods

*E. coli *strain W3110 Kohara and MG1655 (seq) were obtained from the E. Coli Genetic Stock Collection (CGSC # 7167 and # 7740, respectively). Cells were grown in liquid LB medium and then DNA was extracted using DNAeasy Tissue Kit (Qiagen). The CGS results provided by Nimblegen are given in Additional File 1. Regions containing putative mutations were PCR amplified using oligonucleotide primers listed in Additional File 2. They were then purified using Qiaquick (Qiagen) and sequenced using Sanger sequencing. Sequence differences were confirmed by manual examination of the trace data.

## Authors' contributions

CDH prepared samples for CGS, analyzed data and drafted the manuscript. BØP supervised the project and participated in drafting the manuscript.

## Supplementary Material

Additional file 1 Comparative Genome Sequencing results provided by Nimblegen. A description of each file within this bundle is found in the file CGR_Readme.doc Click here for file

Additional file 2 Oligonucleotide primers. A list of the oligonucleotide primers used in this study. Click here for file

## References

[B1] Manjunatha UH, Boshoff H, Dowd CS, Zhang L, Albert TJ, Norton JE, Daniels L, Dick T, Pang SS, Barry CE (2006). Identification of a nitroimidazo-oxazine-specific protein involved in PA-824 resistance in Mycobacterium tuberculosis. Proc Natl Acad Sci U S A.

[B2] Albert TJ, Dailidiene D, Dailide G, Norton JE, Kalia A, Richmond TA, Molla M, Singh J, Green RD, Berg DE (2005). Mutation discovery in bacterial genomes: metronidazole resistance in Helicobacter pylori. Nat Methods.

[B3] Beres SB, Richter EW, Nagiec MJ, Sumby P, Porcella SF, DeLeo FR, Musser JM (2006). Molecular genetic anatomy of inter- and intraserotype variation in the human bacterial pathogen group A Streptococcus. Proc Natl Acad Sci U S A.

[B4] Sumby P, Whitney AR, Graviss EA, DeLeo FR, Musser JM (2006). Genome-wide analysis of group a streptococci reveals a mutation that modulates global phenotype and disease specificity. PLoS Pathog.

[B5] Wong CW, Albert TJ, Vega VB, Norton JE, Cutler DJ, Richmond TA, Stanton LW, Liu ET, Miller LD (2004). Tracking the evolution of the SARS coronavirus using high-throughput, high-density resequencing arrays. Genome Res.

[B6] Zhang W, Qi W, Albert TJ, Motiwala AS, Alland D, Hyytia-Trees EK, Ribot EM, Fields PI, Whittam TS, Swaminathan B (2006). Probing genomic diversity and evolution of Escherichia coli O157 by single nucleotide polymorphisms. Genome Res.

[B7] Zwick ME, McAfee F, Cutler DJ, Read TD, Ravel J, Bowman GR, Galloway DR, Mateczun A (2005). Microarray-based resequencing of multiple Bacillus anthracis isolates. Genome Biol.

[B8] Herring CD, Raghurathan A, Honisch C, Patel T, Applebee MK, Joyce AR, Albert TJ, Blattner FR, van den Boom D, Cantor CR, Palsson BØ (2006). Comparative genome sequencing of Escherichia coli allows bacterial evolution to be observed on a laboratory timescale. Nat Genet.

[B9] Shendure J, Porreca GJ, Reppas NB, Lin X, McCutcheon JP, Rosenbaum AM, Wang MD, Zhang K, Mitra RD, Church GM (2005). Accurate multiplex polony sequencing of an evolved bacterial genome. Science.

[B10] Gresham D, Ruderfer DM, Pratt SC, Schacherer J, Dunham MJ, Botstein D, Kruglyak L (2006). Genome-wide detection of polymorphisms at nucleotide resolution with a single DNA microarray. Science.

[B11] Velicer GJ, Raddatz G, Keller H, Deiss S, Lanz C, Dinkelacker I, Schuster SC (2006). Comprehensive mutation identification in an evolved bacterial cooperator and its cheating ancestor. Proc Natl Acad Sci U S A.

[B12] Margulies M, Egholm M, Altman WE, Attiya S, Bader JS, Bemben LA, Berka J, Braverman MS, Chen YJ, Chen Z, Dewell SB, Du L, Fierro JM, Gomes XV, Godwin BC, He W, Helgesen S, Ho CH, Irzyk GP, Jando SC, Alenquer ML, Jarvie TP, Jirage KB, Kim JB, Knight JR, Lanza JR, Leamon JH, Lefkowitz SM, Lei M, Li J, Lohman KL, Lu H, Makhijani VB, McDade KE, McKenna MP, Myers EW, Nickerson E, Nobile JR, Plant R, Puc BP, Ronan MT, Roth GT, Sarkis GJ, Simons JF, Simpson JW, Srinivasan M, Tartaro KR, Tomasz A, Vogt KA, Volkmer GA, Wang SH, Wang Y, Weiner MP, Yu P, Begley RF, Rothberg JM (2005). Genome sequencing in microfabricated high-density picolitre reactors. Nature.

[B13] Hayashi K, Morooka N, Yamamoto Y, Fujita K, Isono K, Choi S, Ohtsubo E, Baba T, Wanner BL, Mori H, Horiuchi T (2006). Highly accurate genome sequences of Escherichia coli K-12 strains MG1655 and W3110. Mol Syst Biol.

[B14] Kohara Y, Akiyama K, Isono K (1987). The physical map of the whole E. coli chromosome: application of a new strategy for rapid analysis and sorting of a large genomic library. Cell.

[B15] Hacia JG, Edgemon K, Fang N, Mayer RA, Sudano D, Hunt N, Collins FS (2000). Oligonucleotide microarray based detection of repetitive sequence changes. Human Mutation.

[B16] Soupene E, van Heeswijk WC, Plumbridge J, Stewart V, Bertenthal D, Lee H, Prasad G, Paliy O, Charernnoppakul P, Kustu S (2003). Physiological studies of *Escherichia coli *strain MG1655: growth defects and apparent cross-regulation of gene expression. J Bacteriol.

[B17] Cutler DJ, Zwick ME, Carrasquillo MM, Yohn CT, Tobin KP, Kashuk C, Mathews DJ, Shah NA, Eichler EE, Warrington JA, Chakravarti A (2001). High-throughput variation detection and genotyping using microarrays. Genome Res.

[B18] Molla M, Shavlik J, Albert T, Richmond T, Smith S (2004). A self-tuning method for one-chip SNP identification. The proceedings of the IEEE Conference on Computer Systems Bioinformatics.

