# Supplementary material for: An evaluation of Comparative Genome Sequencing (CGS) by comparing two previously-sequenced bacterial genomes
Source: BMC Genomics. 2007 Aug 14;8:274. doi: 10.1186/1471-2164-8-274 (PMC2072959; doi:10.1186/1471-2164-8-274)
Supplement: Additional file 1 — Comparative Genome Sequencing results provided by Nimblegen. A description of each file within this bundle is found in the file CGR_Readme.doc [file 1471-2164-8-274-S1.zip › OID_3998/NimbleGen_data_formats.pdf]

NimbleGen Data Formats

NimbleGen Systems, Inc.

June 15, 2005

Contents

1 Introduction **3**

2 General Information About Array Designs **4**
2.1 Coordinate Information 4
2.2 Containers 4
2.3 Probe Density 4
2.4 Link Placement 5
2.5 Probe Layout 5

3 Array Layout File (NAL) **7**

4 Design File (NDF) **8**

5 Gene Description File (NGD) **11**

6 Positions File (POS) **12**

7 GFF Report (GFF) **14**

8 Feature Report (FTR) **17**

9 PAIR Report (PAIR) **19**

10 NimbleGen XYS Report (XYS) **22**

11 Gene Expression Values (CALLS) **23**

12 Normalized Gene Expression Values (CALLS) **25**

Chapter 1

Introduction

This document gives a description of NimbleGen's various data formats, as well as explanation of array layouts and feature densities. Not all files are relevent to all applications. Most of NimbleGen's data files are tab-delimited text files, with the exceptions noted below. Generally, the columns described for each file will be found in the same order as detailed below, but column order should not be assumed. All of the text data files have one or more header lines and the necessary column information should be parsed out of the header line(s).

Chapter 2

General Information About Array Designs

The flexibility of NimbleGen's array synthesis platform means that there are wide variety of designs that can be created. The following sections provide a brief introduction to NimbleGen array designs, and concepts and nomenclature that are used throughout the rest of the document.

2.1 Coordinate Information

NimbleGen arrays are synthesized using maskless array technology. A digital light projector (DLP[1]) is used to selectively deprotect features where new DNA bases are to be added. The DLP is 768 column x 1204 rows (XVGA). When specifying coordinates, arrays are viewed in portrait, with (1,1) = upper left, (768,1) = upper right, (1,1024) = lower left, (768,1024) = lower right. These are the X and Y coordinates that used in the remainder of the document. The design file also has COL_NUM and ROW_NUM information. These are container-based coordinates, relative to the upper left corner of the container.

2.2 Containers

NimbleGen's array layout application, ArrayScribe, works much the same way as a paint program. Containers, drawn as rectangles or squares on a 'canvas', are positioned by the researcher and then filled by dragging and dropping probe sets on to the containers. Container can be completely overlapping, non-overlapping, partially overlapping, completely contained within one another, etc. They can be 1 x 1 features or 768 x 1024 features. Each container is named, though they do not have to be uniquely named. For example, you could scatter four small containers around the array and label each of them 'CONTROL', or you could label each container separately: 'CONTROL1','CONTROL2', etc. Besides name, position, height, width, and color, each container has several other special properties that can be set. Those include probe density, link placement, and probe layout.

2.3 Probe Density

Probe density deals with how many individual DLP mirrors or elements are used to synthesize a probe, and how those DLP mirrors are arranged. A DLP mirror is 16 microns square, with a 1 micron spacing, and each one creates a feature on the array of the same size (i.e. there is no magnification that takes

[1] `http://www.dlp.com/`

place). By using multiple mirrors, however, larger features can be created called meta-features. If four mirrors instead of one are coordinated together, a meta-feature 33 by 33 microns in size is created (16 + 1 + 16). See Figure 2.1 E for examples of meta-features, which are outlined with thicker black lines. The individual features comprising a meta-features are tied together using a FEATURE_ID in the design file. NimbleScan, NimbleGen's image quantification and data analysis tool, will automatically recognize the presence of meta-features and quantify them as a single feature, rather than quantifying each individual feature separately.

In addition to feature size (how many DLP mirrors are used), the pattern in which they are arranged is important. There are three common feature densities used for NimbleGen arrays: 1:2 (1 in 2), 1:4 (1 in 4) and 4:9 (4 in 9). The first number is the number of occupied features, the second number is the total number of features for the feature cell. For a 1:2 design, there is one blank feature for every occupied feature (see Figure 2.1 A). For a 1:4 probe density, for every 4 features, one is used and there are 3 blank features surrounding it (see Figure 2.1 C). Finally, for a 4:9 feature density, there are 4 occupied features and 5 blank features.

2.4 Link Placement

Expression designs sometimes have mismatch probes - probes that differ from the parent probe by one or more base pair changes at different positions in the probe. ArrayScribe has two options when using mismatch probes, horizontal and vertical. When the horizonal option is selected, the mismatch probe is placed to the right of the perfect match probe. When the vertical option is selected, the mismatch probe is placed directly beneath the perfect match probe. Generally, the vertical option is the default for expression designs with mismatch probes.

2.5 Probe Layout

Probe layout describes how the probes are added when they are dropped on a container. There are four choices: row/column, horizontal, vertical, and random. The row/column option is only used when the probe set(s) contain COL_NUM and ROW_NUM information. It precisely places probes and is generally used for the placement of control probes in defined positions. The horizontal option instructs ArrayScribe to position the probes in horizontal strips, rastering left to right across the container, using the same order as they are found in the probe file. The vertical option does the same, but goes from top to bottom. The random option is the most commonly used option. Probes are placed randomly within the container, though mismatch probes are kept adjacent to their perfect match partners using the link placement specification.

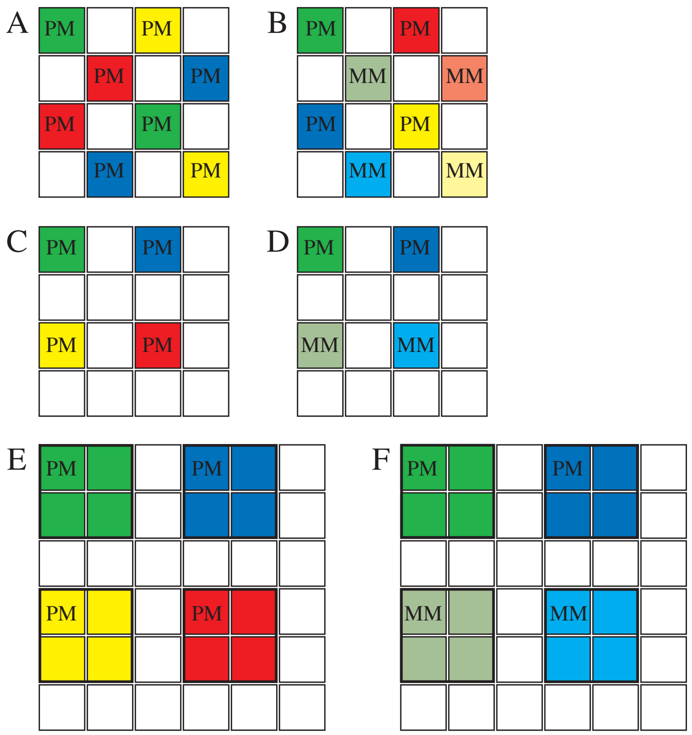

Figure 2.1: (A) 1:2 probe density with no mismatch features. (B) 1:2 probe density with vertical mismatches. (C) 1:4 probe density with no mismatch features. (D) 1:4 probe density with vertical mismatches. (E) 4:9 probe density with no mismatch features. (F) 4:9 probe density with vertical mismatches.

Chapter 3

Array Layout File (NAL)

The NimbleGen Array Layout File (NAL) is the save file format for ArrayScribe, NimbleGen System's array layout application. ArrayScribe allows the user to import probe sets and arrange them into a completed array design. The NAL file stores probe sets and probe layouts. The NAL is a binary file, storing the probe sets in compressed format for more compact storage. Using ArrayScribe, a design file (section 4) and mask sets for array synthesis are produced.

Chapter 4

Design File (NDF)

A design file contains the complete information necessary to synthesis an array.

A design file contains 17 columns. For a standard 1:4 design, the NDF file will contain approximately 196000 rows. For a standard 1:2 design, the NDF file will contain approximately 393000 rows. The information is tab delimited with a single header line containing the field names. The columns can be in any order.

The columns are:

**Field Name**	**Description**	**Requirement**
PROBE_DESIGN_ID	This a unique, composite key consisting of the DESIGN_ID, ROW_NUM and COL_NUM.	Supplied by database
DESIGN_ID	This is the NimbleGen identifier for the design.	Supplied by NimbleGen
CONTAINER	NimbleGen arrays are divided into containers. One method of using containers is to divide the array into quadrants with reference marks. Will be used as 'GENE_EXPR_OPTION' for PAIR and CALLS reports.	Supplied by ArrayScribe. Limited to 50 characters
DESIGN_NOTE	A comment field suitable for placing information necessary for data analysis, for instance if you want to analyze sets of probes and/or genes together that don't separate out using SEQ_ID, PROBE_ID, SELECTION_CRITERIA, CONTAINER, or other information.	Optional - can be used if you want to analyze data using criteria other than CONTAINER (rarely necessary). Limited to 100 characters.
Continued on next page		

Table 4.1 – continued from previous page

**Field Name**	**Description**	**Requirement**
SELECTION_CRITERIA	Generally contains information about how a probe was selected. For rank selection, contains rank, uniqueness, and frequency. For older designs, will contain a criteria category.	Optional - necessary only if you want to evaluate probe sets at different levels of selection criteria. Limited to 100 characters.
SEQ_ID	The NimbleGen sequence identifier. Used to group the probe pairs together for determing gene expression summary values.	Required - must be unique for each sequence/region of interest. Limited to 50 characters. For expression designs, an NGS SEQ_ID is usually 17 character string that looks like HSAP0001S00001834. The first four letters (HSAP) are the species code. The next four characters (0001) are the sequence build number. The 'S' is the designator for sequence (so there's no confusion with the similar looking PROBE_IDs) The last eight digits are the unique sequence number within the sequence build
POSITION	Position of the PROBE_SEQUENCE in the sequence/region of interest, starting from the left/5' end.	Optional - useful for data analysis. Integer
PROBE_SEQUENCE	The DNA sequence synthesized on the array. Always shown 5' to 3'.	Required. Limited to 200 characters
MISMATCH	The mismatch index of the PROBE_SEQUENCE. This will be 0 (for the perfect match probe) , 1 for the first mismatch, 2 for the next, etc.	Required for expression arrays. 0 for perfect match, other positive integer for mismatch. Generated by ArrayScribe. May be overridden by users if MISMATCH in probe file >= 10,000.
MATCH_INDEX	Integer number that ties probe pairs together. Using the combination of MATCH_INDEX and MISMATCH you can retrieve and distinguish the members of the probe pair.	Required for expression arrays with mismatches. Must be unique for each probe pair. Integer. Generated by ArrayScribe. May be over-ridden by users if MATCH_INDEX in probe file >= 1,000,000

Continued on next page

Table 4.1 – continued from previous page

Field Name	Description	Requirement
FEATURE_ID	Unsigned Integer that uniquely identifies a feature. A feature is the set of probes on the array that are to be considered as one entity. A 4:9 array will have 4 probes with the same FEATURE_ID	Integer. Unique within a design for each meta-feature. Generated by ArrayScribe.
COL_NUM	The X or column coordinate of the feature in the CONTAINER.	Integer. Will range from 1 to 768. May be supplied by user if specifying coordinate position.
ROW_NUM	The Y or row coordinate of the feature in the CONTAINER.	Integer. Will range from 1 to 1024. May be supplied by user if specifying coordinate position.
X	The X or column coordinate of the feature in the design.	Integer. Will range from 1 to 768.
Y	The Y or row coordinate of the feature in the design.	Integer. Will range from 1 to 1024.
PROBE_CLASS	Designates probe purpose.	For internal use. Generally 'experimental' or 'control' or 'fiducial'. 'fiducial' is mandatory for those features used for extraction. Limited to 20 characters.
PROBE_ID	The NimbleGen probe identifier. Used to identify a probe sequence within a design.	Limited to 50 characters. For expression designs, a PROBE_ID is a 17 character string that looks like HSAP00P0001724033. The first four letters (HSAP) are the species code. The 'P' is the designator for probe (so there's no confusion with the similar looking SEQ_IDs) The last ten digits are the probe number.

Chapter 5

Gene Description File (NGD)

A gene description file (.ngd) is a tab-delimited file with a header line that contains just 2 columns. The first column is the NimbleGen sequence identifier assigned to the target sequence when loaded into the content database. Depending on the sequence source and type of design, it might be a GenBank accession number, a chromosome and location string (i.e. chrN:start-end), or a NimbleGen identifier as described above for the design file. The second column is a pipe-delimited (“ | ”) string of information that is known about the sequence. Because of the wide variety of sequence sources used for custom and catalog designs, there is no uniform specification for the information contained in this second column. It might be a single piece of information, such as a gene name or description, or it may be a multi-value list containing chromosome position, strand, exon number and positions, etc. The first value of the header line of the NGD file is always SEQ_ID. The second value is a pipe-delimited string that contains the column names of the pipe-delimited values below. Loading the NGD file in an application like Microsoft Excel and splitting the second column on “ | ” will produce a multi-column file with the appropriate column headings. The header for a catalog expression design may look like the following:

SEQ_ID [tab] SEQ_UNIQUE | SPECIES_CODE | BUILD | FEAT_TYPE | GENE_NAME | ACCESSION | GI | FUNCTION | CHROMOSOME | MAPLOC | DESCRIPTION | COMMENTS | DATE_ENTERED | SOURCE_DB [eol]

Generally the column headings have informative names that should be simple to interpret.

Chapter 6

Positions File (POS)

The positions file (.pos) is used for applications like comparative genomic hybridization (CGH), expression tiling, or ChIP-chip, where it is essential to know the genomic position of the probes used in the experiment. The .pos file contains the mapping of each PROBE_ID to its position in the genome. Positions are keyed on SEQ_ID plus PROBE_ID, so the PROBE_IDs do not have to be unique, though that is generally a good idea. Multiple versions of a .pos file may exist for a design, representing different genomic builds, or designating different ways of displaying the data in SignalMap, NimbleGen's visualization tool. For example, if a design encompasses 20 different genomic regions, the researcher may want to see the data displayed at the actual genomic coordinates, or may want the data for each region displayed in a separate panel. POS files have 4 required columns and a number of optional columns. They are tab-delimited files with a single header line. The columns may be in any order.

The columns are:

Field Name	Description	Notes
PROBE_ID	The NimbleGen probe identifier. For genomic applications this is most often in the form of [chromosome]00P[position]. For example, CHR0100P000053157 or CHRY00P057369061. The "00P" string indicates the probe was generated from the forward strand. A "99P" string indicates the probe is the reverse complement of the probe generated from that postion on the forward strand.	Required. Generally all uppercase, with zero padding for chromosome name. Limited to 50 characters.

Continued on next page

Table 6.1 – continued from previous page

Field Name	Description	Notes
SEQ_ID	The NimbleGen sequence identifier. For genomic applications this is normally either the chromosome name (CHR1,CHRY) or the chromosome name with a position qualifier in the format chrN:start-stop (CHR1:1-10000000, CHRY:2300000-7800000).	Required. Generally all upper case, with no zero padding for chromosome name. Limited to 50 characters.
CHROMOSOME	The chromosome or parent sequence that the probe is located on. May be different from the chromosome indicated by SEQ_ID and/or PROBE_ID because file was generated against different genome build, or different set of target sequences.	Required. Generally lower case, with no zero padding. Limited to 50 characters.
POSITION	Position of the PROBE_SEQUENCE in the sequence/region of interest, starting from the left/5' end.	Required. Integer
COUNT	The uniqueness count of the probe in the target genome. Different applications may have different requirements for uniqueness. For designs where all probes are equal length, the COUNT will be the number of matches of that length in the genome. For designs with variable length probes, uniqueness is normally measured as the number of matches equal to the size of the shortest allowed probe on the design. May default to 1 for some applications.	Optional column. Integer
LENGTH	Length of the probe in base pairs.	Optional column. Integer
GC	The percent GC of the probe sequence.	Optional column. Float

Chapter 7

GFF Report (GFF)

General Feature Format (GFF) is an exchange format for genomic based data. NimbleGen products that contain genomic coordinates and information supply data in GFF format for viewing in SignalMap, NimbleGen's visualization tool, and in other GFF viewers. GFF files supplied by NimbleGen conform to version 2 of the GFF specification [1]. Each line of a GFF file is tab-delimited, with the following format:

<seqname> <source> <feature> <start> <end> <score> <strand> <frame> [attributes] [comments]

The columns are:

**Field Name**	**Description**	**Notes**
seqname	The name of the sequence. Having an explicit sequence name allows a feature file to be prepared for a data set of multiple sequences. Normally the seqname will be the identifier of the sequence in an accompanying fasta format file. An alternative is that ¡seqname¿ is the identifier for a sequence in a public database, such as an EMBL/Genbank/DDBJ accession number. Which is the case, and which file or database to use, should be explained in accompanying information.	For NimbleGen data this will normally be the chromosome name. This is the panel name in SignalMap.
source	The source of this feature. This field will normally be used to indicate the program making the prediction, or if it comes from public database annotation, or is experimentally verified, etc.	Will generally be NimbleGen program that produced the data, or the database name.

Continued on next page

[1] http://www.sanger.ac.uk/Software/formats/GFF/GFF_Spec.shtml

Table 7.1 – continued from previous page

**Field Name**	**Description**	**Notes**
feature	The feature type name.	For NimbleGen data, this will often be the sample name or some description of the values. This will be the track name in SignalMap.
start, end	Integers. **start** must be less than or equal to **end**. Sequence numbering starts at 1, so these numbers should be between 1 and the length of the relevant sequence, inclusive. Version 2 condones values of **start** and **end** that extend outside the reference sequence. This is often more natural when dumping from acedb, rather than clipping. It means that some software using the files may need to clip for itself.	These are the genomic coordinates of the feature.
score	A floating point value. When there is no score (i.e. for a sensor that just records the possible presence of a signal, as for the EMBL features above) you should use '.'.	This score will have various meanings, depending on the NimbleGen product. For CGH and ChIP-chip, it might be log2 values for some tracks. It might be probabilities or false discovery rates for other tracks. Use the 'feature' identifier to provide some indication of the meaning of the score.
strand	One of '+', '-' or '.'. '.' should be used when strand is not relevant, e.g. for dinucleotide repeats. Version 2 change: This field is left empty '.' for RNA and protein features.	Generally only relevant for gene annotation information.

Continued on next page

Table 7.1 – continued from previous page

Field Name	Description	Notes
frame	One of '0', '1', '2' or '.'. '0' indicates that the specified region is in frame, i.e. that its first base corresponds to the first base of a codon. '1' indicates that there is one extra base, i.e. that the second base of the region corresponds to the first base of a codon, and '2' means that the third base of the region is the first base of a codon. If the strand is '-', then the first base of the region is value of **end**, because the corresponding coding region will run from **end** to **start** on the reverse strand. As with **strand**, if the frame is not relevant then set **frame** to '.'. Version 2 change: This field is left empty '.' for RNA and protein features.	Generally only relevant for gene annotation information.
attribute	From version 2 onwards, the attribute field must have an tag value structure following the syntax used within objects in a .ace file, flattened onto one line by semicolon separators. Tags must be standard identifiers ([A-Za-z][A-Za-z0-9_]*). Free text values must be quoted with double quotes. Note: all non-printing characters in such free text value strings (e.g. newlines, tabs, control characters, etc) must be explicitly represented by their C (UNIX) style backslash-escaped representation (e.g. newlines as '\n', tabs as '\t'). As in ACEDB, multiple values can follow a specific tag.	NimbleGen uses the attribute field to specify URLs to external databases and to specify feature colors.

Chapter 8

Feature Report (FTR)

The Feature Report provides the signal intensity for every non-blank feature on the array as an average of the pixel intensities comprising the each feature. Meta-features are not aggregated in this report - each individual feature which is part of a meta-feature is reported separately.
The information in a feature report is tab delimited with a two header lines. The first header line contains information in key=value pairs regarding the originating image, design, and grid placement parameters. The second header line contains the column names. A feature report contains 11 columns. The columns are:

**Field Name**	**Description**	**Notes**
X	The X or column coordinate of the feature in the design.	Integer. Will range from 1 to 768.
Y	The Y or row coordinate of the feature in the design.	Integer. Will range from 1 to 1024.
SEQ_ID	The NimbleGen sequence identifier. Used to group the probe pairs together for determing gene expression summary values.	For expression designs, an NGS SEQ_ID is usually 17 character string that looks like HSAP0001S00001834. The first four letters (HSAP) are the species code. The next four characters (0001) are the sequence build number. The 'S' is the designator for sequence (so there's no confusion with the similar looking PROBE_IDs) The last eight digits are the unique sequence number within the sequence build

Continued on next page

Table 8.1 – continued from previous page

**Field Name**	**Description**	**Notes**
PROBE_ID	The NimbleGen probe identifier. Used to identify a probe sequence within a design.	For expression designs, a PROBE_ID is a 17 character string that looks like HSAP00P0001724033. The first four letters (HSAP) are the species code. The 'P' is the designator for probe (so there's no confusion with the similar looking SEQ_IDs) The last ten digits are the probe number.
X_PIXEL	X coordinate of the upper left corner of the feature in image coordinates	
Y_PIXEL	Y coordinate of the upper left corner of the feature in image coordinates	
HEIGHT	Height of the feature in pixels	Will depend on the feature size and the scanner resolution.
WIDTH	Width of the feature in pixels	Will depend on the feature size and the scanner resolution.
FGD_PIX	Abbreviation for foreground pixels. Total number of pixels in the feature.	
SIGNAL_MEAN	Mean fluorescence intensity of the pixels which make up the feature.	Will range from 0 to 65535.
SIGNAL_STDEV	Standard deviation of the fluorescence intensity of the pixels which make up the feature.	

Chapter 9

PAIR Report (PAIR)

The PAIR report is the raw data file format for a number of NimbleGen products. Meta-features are pre-aggregated in a PAIR report. For designs without meta-features, a PAIR and FTR report will contain essentially the same information.
A PAIR report contains 11 columns. The information is tab delimited with a two header lines. The first header line contains information in key=value pairs regarding the originating image, design, and grid placement parameters. The second header line contains the column names. The columns can be in any order. May also be named *_pair.txt.

The columns are:

Field Name	Description	Notes
IMAGE_ID	The name of the image the data was extracted from, minus the .tif extension	For NimbleGen data sets, this wil be the array identifier plus any additional information, like wavelength used to scan the array, or photomultiplier tube setting. The array ID will be all of the characters before the first underscore.
GENE_EXPR_OPTION	The CONTAINER name from the design file, if analysis was done 'by container' or WHOLE_ARRAY if all replicate probe sets were combined into a single set.	The default analysis is normally 'by container' . CONTAINER names are generally name FORWARD/REVERSE, BLOCK1/BLOCK2/etc. or other similar conventions.
Continued on next page		

Table 9.1 – continued from previous page

Field Name	Description	Notes
SEQ_ID	The NimbleGen sequence identifier. Used to group the probe pairs together for determing gene expression summary values.	Required - must be unique for each sequence/region of interest. Limited to 50 characters For expression designs, an NGS SEQ_ID is usually 17 character string that looks like HSAP0001S00001834. The first four letters (HSAP) are the species code. The next four characters (0001) are the sequence build number. The 'S' is the designator for sequence (so there's no confusion with the similar looking PROBE_IDs) The last eight digits are the unique sequence number within the sequence build
PROBE_ID	The NimbleGen probe identifier. Used to identify a probe sequence within a design.	Limited to 50 characters. For expression designs, a PROBE_ID is a 17 character string that looks like HSAP00P0001724033. The first four letters (HSAP) are the species code. The 'P' is the designator for probe (so there's no confusion with the similar looking SEQ_IDs) The last ten digits are the probe number.
POSITION	Position of the PROBE_SEQUENCE in the sequence/region of interest, starting from the left/5' end.	Optional - useful for data analysis. Integer
X	The X or column coordinate of the feature in the design.	Integer. Will range from 1 to 768. For aggregate features, made up of multiple features, this coordinate is the coordinate of the upper left feature. For probe pairs, the same coordinates are used for both the perfect match and mismatch feature since they are always physically adjacent.

Continued on next page

Table 9.1 – continued from previous page

Field Name	Description	Notes
Y	The Y or row coordinate of the feature in the design.	Integer. Will range from 1 to 1024. For aggregate features, made up of multiple features, this coordinate is the coordinate of the upper left feature. For probe pairs, the same coordinates are used for both the perfect match and mismatch feature since they are always physically adjacent.
MATCH_INDEX	Integer number that ties probe pairs together. Using the combination of MATCH_INDEX and MISMATCH you can retrieve and distinguish the members of the probe pair.	Required for expression arrays with mismatches. Must be unique for each probe pair of a given SEQ_ID. Integer.
SEQ_URL	When populated, URL to sequence information for the SEQ_ID.	
PM	The perfect match signal intensity for the probe pair.	Will range from 0 to 65536.
MM	The mismatch signal intensity for the probe pair.	Will range from 0 to 65536. Will be zero for perfect match only designs.

Chapter 10

NimbleGen XYS Report (XYS)

The NimbleGen XYS file format is meant as a minimal data exchange format, containing only 4 columns of data. The individual features comprising a meta-feature are not reported separately, but are instead aggregated and a single value is reported. For every FEATURE_ID in a design file, there is a single row in the XYS file.
The information is tab delimited with a two header lines. The first header line contains information in key=value pairs regarding the originating image, design, and grid placement parameters. The second header line contains the column names.

The columns are:

**Field Name**	**Description**	**Notes**
X	The X or column coordinate of the feature in the design.	Will range between 1 and 768
Y	The Y or row coordinate of the feature in the design.	Will range between 1 and 1024
Signal	The mean fluorescence intensity of the pixels comprising the feature	Will range from 0 to 65535. Is set to NA for NimbleGen control features not related to the experiment.
Count	The number of individual features aggregated for this row of data.	For 1:2 and 1:4 feature densities, this number will be 1. For 4:9 feature densities, this number will be 4. Is set to NA for NimbleGen control features not related to the experiment.

Chapter 11

Gene Expression Values (CALLS)

There are two types of CALLS files. The first type of CALLS file is for gene expression summaries of un-normalized data, and can be found on the path AuxillaryData\GeneExpressionValues\Calls on media deliverables for expression studies. If the array design has mismatches, these files are the ones that should be used to examine gene expression values where the mismatch probe.

The CALLS files contains a gene expression summary value for each gene in an expression array. If there are replicate probe sets, where may be multiple values for each gene, one for each replicate. The GENE_EXPR_OPTION will contain the CONTAINER name from the design if there are replicate probe sets, or WHOLE_ARRAY if there are no replicates, or if the replicated sets were analyzed as a single probe set.

The columns are:

Field Name	Description	Notes
IMAGE_ID	The name of the image the data was extracted from, minus the .tif extension	For NimbleGen data sets, this wil be the array identifier plus any additional information, like wavelength used to scan the array, or photomultiplier tube setting. The array ID will be all of the characters before the first underscore.
SEQ_ID	The NimbleGen sequence identifier. Used to group the probe pairs together for determing gene expression summary values.	
PROBE_PAIRS	The number of probe pairs for this SEQ_ID and CONTAINER\GENE_EXPR_OPTION combination.	
FILTERED_PROBE_PAIRS	The number of probe pairs for this SEQ_ID and CONTAINER\GENE_EXPR_OPTION combination after filtering outliers.	Outliers are those probe values that are greater than 3 standard deviations from the mean of the probe set.

Continued on next page

Table 11.1 – continued from previous page

Field Name	Description	Notes
PM_AVG	The gene expression summary value for the gene calculated as the mean of the signal intensities of the perfect match probes only.	Values are in linear scale, though older version of this file might contain log2 values.
PM_CV	The coefficient of variation (CV) of the perfect match probes.	CV is calculated as the standard deviation of the values divided by the mean.
MM_AVG	The mean signal intensity of the mismatch probes only.	Values are in linear scale, though older version of this file might contain log2 values.
MM_CV	The coefficient of variation (CV) of the mismatch probes.	CV is calculated as the standard deviation of the values divided by the mean.
DIFF_AVG	The mean signal intensity of the perfect match probes after subtracting the signal of the mismatch probes.	Values are in linear scale, though older version of this file might contain log2 values. DIFF_AVG may not equal to PM_AVG - MM_AVG because DIFF_AVG is calculated as the mean of the differences, and there may be rounding differences.
DIFF_CV	The coefficient of variation (CV) of the values of the perfect match signal minus the mismatch signal.	CV is calculated as the standard deviation of the values divided by the mean.
GENE_EXPR_OPTION	The CONTAINER name from the design file, if analysis was done 'by container' or WHOLE_ARRAY if all replicate probe sets were combined into a single set.	The default analysis is normally 'by container' . CONTAINER names are generally name FORWARD/REVERSE, BLOCK1/BLOCK2/etc. or other similar conventions.

Chapter 12

Normalized Gene Expression Values (CALLS)

The second type of CALLS file is for gene expression summaries of normalized data, and can be found on the path AuxillaryData\NormalizedData\Calls on media deliverables for expression studies. The gene expression values in these files have been produced using the RMA (Robust Multichip Average) algorithm. There is a gene expression summary value for each gene in an expression array. If there are replicate probe sets, where may be multiple values for each gene, one for each replicate. The GENE_EXPR_OPTION will contain the CONTAINER name from the design if there are replicate probe sets, or WHOLE_ARRAY if there are no replicates, or if the replicated sets were analyzed as a single probe set.

The columns are:

**Field Name**	**Description**	**Notes**
IMAGE_ID	The name of the image the data was extracted from, minus the .tif extension	For NimbleGen data sets, this wil be the array identifier plus any additional information, like wavelength used to scan the array, or photomultiplier tube setting. The array ID will be all of the characters before the first underscore.
SEQ_ID	The NimbleGen sequence identifier. Used to group the probe pairs together for determing gene expression summary values.	
EXPRS	The gene expression summary value for the gene.	Values are in linear scale, though older version of this file might contain log2 values.
GENE_EXPR_OPTION	The CONTAINER name from the design file, if analysis was done 'by container' or WHOLE_ARRAY if all replicate probe sets were combined into a single set.	The default analysis is normally 'by container' . CONTAINER names are generally name FORWARD/REVERSE, BLOCK1/BLOCK2/etc. or other similar conventions.
