# Supplementary material for: An evaluation of Comparative Genome Sequencing (CGS) by comparing two previously-sequenced bacterial genomes
Source: BMC Genomics. 2007 Aug 14;8:274. doi: 10.1186/1471-2164-8-274 (PMC2072959; doi:10.1186/1471-2164-8-274)
Supplement: Additional file 1 — Comparative Genome Sequencing results provided by Nimblegen. A description of each file within this bundle is found in the file CGR_Readme.doc [file 1471-2164-8-274-S1.zip › OID_3998/CGR_Readme.doc]

**NimbleGen Comparative Genomic Resequencing (CGR) readme.txt file**

This file explains the data output from the CGR process.

**Genome Mapping files include:**

1. <Samplename>_ratio.txt

2. <Samplename>_ratio.gff

**Genome Resequencing files include:**

1. <Samplename>_snp_info.txt

2. <Samplename>_reference_seqs.aa

3. <Samplename>_reference_seqs.fas

4. <Samplename>_snp_seqs.aa

5. <Samplename>_snp_seqs.fas

All files except the <Samplename>_ratio.gff are text files and can be opened with text editors. The <Samplename>_snp_info.txt file is best viewed in Microsoft Excel or other spreadsheet programs.

**1. <Samplename>_ratio.txt**

This file contains the raw probe intensity and intensity ratio data used for the mapping portion of the experiment. If resequencing has been preformed, it will also contain SNP and non-called ROI (regions of interest) data. The file contains the following columns:

SEQ_ID: The name of the sequence being analyzed. It is often the Genbank accession number. There may be more than one SEQ_ID in this column, such as one for the chromosome and one for each plasmid.

POSITION: The start position of the probe in the genome. Probes are typically 29mers for genomes with an average G/C content >=40%, and 32mers for genomes <40% G/C. The spacing is typically 7 or 8 base spacing from the start of one probe to the start of the next. This number can be higher for lower resolution mapping, or higher for very high-resolution mapping.

RATIO: The ratio of the reference probe intensity divided by the test probe intensity. Reference probes and test probes that do not span a mutation should represent full-length perfect match hybridization, and thus should have similar intensities, with a reference/test ratio near 1. Probes that span mutations in the test genome typically display lower hybridization intensities than the corresponding reference probes, and thus the reference/test ratio will shift above 1. If the test genome contains an amplification event (increased copy number when compared to the reference), then the reference/test ratio will shift below 1.

Note: If the reference genome used to design the arrays is different from the actual reference used in the experiment, the reference probe intensity values may not represent full-length perfect match hybridization signal. These regions may not provide enough signal to be suitable for robust data generation.

DIFFERENCES: This column displays a 0 if a probe intensity ratio (reference/test) does not deviate significantly from 1. If the intensity ratio is significantly above one, then the column will contain a 1. Ratio shifts that are significantly below 1 (indicating an increase in copy number in the test genome) are not automatically flagged, but can be visualized when viewing the <Samplename>_ratio.gff file, as described below.

This column will not be present if resequencing was performed. The following two columns will replace it.

SNP: If resequencing has been performed, this column indicates whether a probe spans a position that the resequencing arrays called a SNP. Y indicates the probe spans at least one SNP, N indicates no SNPs were identified in this region of the genome.

NON_CALLED_ROI: If resequencing was performed, this column indicates probes that had a reference/test intensity ratio significantly above 1, but were not called as a SNP by resequencing. These positions, indicates by a Y, may represent non-SNP mutations, such as deletions, sites of insertion, the end points of inversions or translocations, or SNPs that were not called reliably by resequencing. The exact nature of these positions can be obtained by other techniques, such as PCR amplification followed by capillary sequencing. Ratio shifts that are significantly below 1 (indicating an increase in copy number in the test genome) are not automatically flagged, but can be visualized when viewing the <Samplename>_ratio.gff file, as described below.

AVE_REF: The average of the forward and reverse probe intensities from the reference genome hybridization.

AVE_TEST: The average of the forward and reverse probe intensities from the test genome hybridization.

RUN_LENGTH: The number of probes in a row that have a reference/test intensity ratio that deviates significantly from one. This is useful for identifying larger deletions or hypervariable regions in the genome. Isolated SNPs will typically not have a run count of >5 at 7 to 8 base probe spacing, so runs larger than this are indicative of other larger genomic events.

**2. <Samplename>_ratio.gff**

The <Samplename>_ratio.gff file is designed to be viewed using NimbleGen’s SignalMap visualization data. This viewer allows the data from the entire genome to be easily browsed. It contains data “tracks” that indicate the positions of SNPs and other potential mutations, as well as raw data. A users manual for SignalMap (in PDF format) is available from NimbleGen if one was not delivered with the Software. The following data tracks may or may not be present, depending on the availability of genome annotation and resequencing information:

Genome Annotation (generally named with the genome identifier, typically the Accession number if available): Displays colored blocks representing annotated genes. Blocks above the line indicate genes encoded on the forward strand of the genome, while bars below the line represent genes encoded on the reverse strand. The CDS data field from the Genbank annotation file is used to generate this track. If the Genbank file was annotated with hyperlinks, double-clicking on a gene block will open a web browser and target the NCBI database for known information about the selected gene. If no Genbank annotation file was available at the time of analysis, this track will be absent.

DIFFERENCES_<samplename>: Displays positions in the genome that have a significant ratio shift between reference and test probes for both strands. These positions may represent SNP, deletions, sites of insertion, the end points of inversions or translocations. It is sometimes possible to distinguish mutation signatures. Deletions tend to have large ratio shifts, while SNP ratio shifts tend to be subtler. The number of consecutive probes shifted by any mutation will allow for an estimation of its size, based on the mapping probe spacing. Most of the mutations represented by SNPs can be resolved by performing follow up resequencing array analysis, as long as the genomes do not appear too divergent (>0.5%).

SNP_<samplename>: This track is only displayed if resequencing was performed. Displays colored coded points or vertical bars that indicate the position of SNPs (base substitutions) identified in the genome by resequencing. If the data is displayed as points, it is highly recommended that the track style is changed to bars, by right clicking on the left side of the y-axis for this track, selecting style, and clicking on bars. This will make the SNP positions much easier to visualize. SNP positions are colored light blue if they fall between annotated genes, green if they fall inside a gene but does not change the amino acid sequence of the encoded protein (synonymous mutation), and dark blue if the SNP changes the amino acid sequence of the encoded protein (non-synonymous mutation). Holding the cursor over the SNP position will display the nucleic acid and amino acid changes at the top of the screen.

NON_CALLED_RIO_<samplename>: This track is only displayed if resequencing was performed. Displays positions in the genome that have a significant ratio shift between reference and test probes for both strands, but were not called as SNPs by the resequencing. These positions may represent non-SNP mutations, such as deletions, sites of insertion, the end points of inversions or translocations, tightly clustered SNPs, or individual SNPs that were not called reliably by resequencing. The number of consecutive probes shifted by any mutation will allow for an estimation of its size, based on the mapping probe spacing. The exact nature of these positions can be obtained by other techniques, such as PCR amplification followed by capillary sequencing.

RESEQUENCED_<samplename>: This track is only displayed if resequencing was performed. Displays positions in the genome that were resequenced using resequencing arrays.

RATIO_<samplename>: Plot of the probe intensity ratio (Reference/test). Reference probes and test probes that do not span a mutation should represent full-length perfect match hybridization, and thus should have similar intensities, with a reference/test ratio near 1. Probes that span mutations in the test genome typically display lower hybridization intensities than the corresponding reference probes, and thus the reference/test ratio will shift above 1. If the test genome contains an amplification event (increased copy number when compared to the reference), then the reference/test ratio will shift below 1.

Note: If the reference genome used to design the arrays is different from the actual reference used in the experiment, the reference probe intensity values may not represent full-length perfect match hybridization signal. These regions may not provide enough signal to allow robust generation of data.

AVE_TEST_<samplename>: Plot of the average of the forward and reverse probe intensities from the test genome hybridization.

AVE_REFERENCE_<samplename>: Plot of the average of the forward and reverse probe intensities from the reference genome hybridization.

**The following files are only generated if resequencing is performed, and a GenBank annotation file is available that matches the sequence used to create the arrays.**

**3. <Samplename>_snp_info.txt**

The snp_info file provides information on SNPs that were identified in the genome. This file is best viewed in a spreadsheet program such as Microsoft Excel. The data columns produced by the SNP analysis are provided below.

SEQ_ID: The genome identifier, typically the Accession number if available.

GENOME_POSITION: The genomic position of each SNP.

REF: The reference nucleotide of each SNP.

SNP: The mutant nucleotide of each SNP.

CONFIDENCE: A composite of several parameters to determine the likelihood that any SNP is real (not a false positive). This score is based on the quality of the SNP call and the similarity of the sequence flanking the SNP to other sequences in the genome that may cross hybridize. SNPs with HIGH confidence scores have a high probability of being true SNPs. LOW confidence scores generally result from failed uniqueness testing, and have a very high likelihood of being false positive base calls. These sites should generally be ignored. SNPs with MEDIUM confidence should be validated by capillary sequencing before biological conclusions are drawn.

PROBABILITY: A relative measure of the quality of a SNP call. The score typically ranges between 1.0 and 0.9. The lower the probability score, the less likely a SNP is to be real. This criterion alone should not be used to assign SNP calls. Called SNP loci that are not unique in the genome often have excellent probability scores but may represent a false positive.

TYPE: Lists whether or not a SNP is found within an annotated gene, or between annotated genes. SNPs in genes are designated as coding, and are listed first. SNPs between genes are designated as intergenic, and are listed second.

NMER_SCORE: A weighted measure of how often perfect matches for increasing numbers of central bases in each probe are found in the genome is calculated: the “Nmer frequency”. Each such perfect match contributes to the Nmer frequency score, with shorter oligos contributing less than longer oligos according to the following equation:

Nmer frequency score = Σ [N-mer frequency]*[0.75^((29mer-Nmer length)/2)]

The sum of the frequency scores for central Nmers from 19, 21, 23, 25, 27, and 29mers is calculated for each Probe calling a SNP. The higher the Nmer frequency score, the more likely it is that the probe will also hybridize elsewhere in the genome. Any SNP being called by a probe with an Nmer frequency above zero is considered a likely false positive.

UNIQUE: The second uniqueness test calculates a “sequence dissimilarity” score is calculated by comparing the 29 bases spanning the SNP to every possible 29 base window in the reference genome. Mismatches at the end of a probe tend to disrupt hybridization less than centrally located mismatches, and hence are weighted differently. The weight value vs. position used in the current study is listed below:

| 1 | 2 | 3 | 4 | 5 | 6 | 7 | 8 | 9 | 10 | 11 | 12 | 13 | 14 | 15 | 16 | 17 | 18 | 19 | 20 | 21 | 22 | 23 | 24 | 25 | 26 | 27 | 28 | 29 |
| --- | --- | --- | --- | --- | --- | --- | --- | --- | --- | --- | --- | --- | --- | --- | --- | --- | --- | --- | --- | --- | --- | --- | --- | --- | --- | --- | --- | --- |
| 1 | 1 | 1 | 2 | 2 | 2 | 2 | 4 | 4 | 4 | 4 | 4 | 4 | 4 | 0 | 4 | 4 | 4 | 4 | 4 | 4 | 4 | 2 | 2 | 2 | 2 | 1 | 1 | 1 |

The dissimilarity score for each possible 29mer in the genome is calculated by adding up the weight vectors for each base not matching its corresponding base in the 29 bases surrounding a SNP. If the lowest dissimilarity score for every 29mer in the genome is greater than 10, the SNP position is considered unique. For example, if the 29mer that most closely matches the 29 bases surrounding a SNP contained 3 mismatches between positions 8 and 22, that SNP position would have a dissimilarity score of 12 (4+4+4), and be sufficiently dissimilar to be considered unique, whereas six mismatches in positions 1 to 6 would not meet the uniqueness threshold of 10 (1+1+1+2+2+2 = 9). SNP sites that pass this criterion (threshold value >10) are given a score of 1, while SNP sites that fail (threshold value <10) are given a score of 0. SNP sites that fail this criterion are considered likely false positives.

HIGHEST_RANK: A measure of how strongly the SNP position was indicated by the mapping data. Ranks above 10,000 are strongly indicated by both strands, whereas numbers below 10,000 were only mapped by one probe. Occasionally, true SNPs are identified by resequencing that were not strongly indicated by mapping.

FORWARD: The average probability score of the five base calls in either direction from the SNP on the forward strand. This is a measure of the SNP “neighborhood” quality.

REVERSE: The average probability score of the five base calls in either direction from the SNP on the reverse strand. This is a measure of the SNP “neighborhood” quality.

FORWARD_INTENSITY: The raw intensity of the probe that called the SNP on the forward strand.

REVERSE_INTENSITY: The raw intensity of the probe that called the SNP on the reverse strand.

AA CHANGE: Categorizes coding SNPs base on whether they change the amino acid sequence of a protein, or not. SYN indicates synonymous SNPs (no amino acid change). NON indicates non-synonymous SNPs (altered amino acid).

ORIG_AA: The amino acid associated with the reference sequence for the corresponding SNP position.

SNP_AA: The amino acid associated with the test sequence, for the corresponding SNP position.

GENE_POSITION: The nucleic acid position mutated within a gene.

LOCUS_TAG: The locus tag field from the Genbank annotation file.

START: The genomic start position of the mutated gene.

END: The genomic end position of the mutated gene.

STRAND: The strand on which the mutated gene is located in the genome.

PRODUCT: The annotation field from the Genbank file.

NOTE: The note field from the Genbank file.
